# Personalized Teaching Questioning Strategies Study Based on Learners’ Cognitive Structure Diagnosis

**DOI:** 10.3390/bs13080660

**Published:** 2023-08-08

**Authors:** Yuan Zhao, Tao Huang, Han Wang, Jing Geng

**Affiliations:** 1 Faculty of Artificial Intelligence in Education, Central China Normal University, Wuhan 430079, China; zhyuan@mails.ccnu.edu.cn (Y.Z.); gengjing@mails.ccnu.edu.cn (J.G.); 2 Faculty of Education, Jiujiang University, Jiujiang 332005, China; 3 School of Education, Hubei University, Wuhan 430062, China; wangh@mails.ccnu.edu.cn

**Keywords:** cognitive structure, personalized teaching questioning, cognitive structure diagnosis, questioning strategy

## Abstract

Personalized education has been a widely shared goal pursued by Chinese and foreign educators. As the primary method of teacher–student interaction, the importance of personalized questioning is self-evident. Due to a lack of technical support, teachers rely on their teaching experience to ask questions without considering the learning situation of learners. This results in teaching questioning being unable to support learners’ learning. These questions are relatively shallow and cannot promote the construction and transfer of learners’ knowledge. Cognitive diagnostic technology could diagnose learners’ cognitive states and provide services for personalized teaching. Therefore, a personalized teaching questioning strategy based on learners’ cognitive structure diagnosis was proposed in this study. Firstly, we diagnosed learners’ cognitive structure through usability, distinguishability, and stability. Secondly, we discussed the types of questions that teachers should raise when facing learners in different situations. We also discussed the application of personalized teaching questioning strategies. The experiment took place at M Primary School in Ningxia, China, with the participation of one teacher and ninety-seven fourth-grade students. Seven lessons were observed and videotaped across a range of topics. The study revealed that personalized teaching questioning strategies could improve learners’ academic performance and subject literacy. They can also increase the number of teacher questioning, change the depth of teacher questioning content, and expand the scope of questioning subjects.

## 1. Introduction

Personalized education delivers effective teaching based on learners’ differences in individual characteristics such as learning styles and preferences, ability levels, and interests. Research on the importance of questioning as a teaching and learning strategy is well documented [1,2,3]. Educators generally believe that effective questioning fosters interaction between teachers and students, while facilitating students’ understanding of concepts taught in the classroom [4,5]. Personalized teaching questioning (PTQ) refers to respecting and paying attention to learners’ differences, asking timely questions appropriate to their ability levels, and promoting the full development of their abilities. Using the standard classroom transaction model of initiation-response-evaluation (IRE), teachers assess where students are so that they can plan the next steps in the learning and teaching processes [6,7]. The traditional method of questioning in the classroom has faced criticism. Teachers who fail to accurately assess the cognitive level of their students before asking questions may make incorrect decisions regarding the duration, depth, and subject matter of their questions. Relevant studies have shown that questioning depth should adapt to students’ cognitive level [6,8]. Cognitive diagnostic techniques are utilized to evaluate a learner’s mental condition precisely. Prior research has focused more on students’ mastery level, ability, and literacy, neglecting the description of learners’ knowledge structure. This is not conducive to achieving personalized teaching questioning. This study proposes personalized teaching questioning strategies based on diagnosing learners’ cognitive structure.

The study aimed to explore personalized teaching questioning strategies based on the learner’s cognitive structure diagnosis. Two research questions set the parameters for this study: 

Q1. What are personalized teaching questioning strategies for diagnosing the learner’s cognitive structure?

Q2. How effective is the personalized teaching questioning strategy based on the learner’s cognitive structure diagnosis?

## 2. Literature Review

### 2.1. Teacher Questioning

As one of the most basic methods of promoting teacher–student interaction and communication, classroom questioning has become increasingly important in the classroom [9]. Research has shown that teachers ask a high frequency of questions. Although there has been an increasing emphasis on the role of students’ questions in learning science [1,2,3], teacher questioning remains a critical component in the classroom. Floyd [10] developed a study with 40 elementary teachers and found that these teachers asked 93 percent of all classroom questions. More recently, Kerry [11] reinforced these numbers, posing that if teachers ask an average of 43.6 questions per hour, they are likely to ask about 2 million questions in an average career. A hierarchal approach to cognition was originally described by Bloom and subsequently modified by Anderson and Krathwohl [12]. According to Anderson and Krathwohl’s taxonomy, problems that elicit responses at the knowledge, comprehension, and application levels are considered low or low-level cognitive problems, whereas problems at the analysis, synthesis, and evaluation levels are considered high or high-level cognitive problems [13,14,15,16]. For example, after teaching about animals that change color, the teacher then asks “Which animals are chatoyant?”; this question requires learners to recall the acquired information. This is a low-level question. “What types of problems might chatoyant animals face if they lost this characteristic?” is a high-level question. For such a question, students are provided to know the chatoyant animals and their characteristics, analyze their wildlife conditions, and contribute initial opinions. Even if teachers ask many questions per class, observations of classroom-based instructors have repeatedly shown that lower-level questions are far more frequently used [17,18]. Higher-order questions elicit deeper and more critical thinking; therefore, teachers are expected to ask higher-level questions for higher-level learning [13]. This does not mean that lower-order questions should not be asked. According to Fisher and Frey [19], teachers should not exclude knowledge, understanding, or application questions that provide students with factual information to solve complex problems. Effective questioning strategies suggest that a question’s depth should match a learner’s cognitive level [8]. Several studies have explored the relationship between questioning time and questioning depth, believing that during lessons, teachers are more likely to raise high-level questions [20,21]. However, this strategy has not yet been explored in detail due to technological limitations.

Recent research also highlighted the value of questioning as an essential teaching and learning tool [22]. Teacher questions serve various purposes and provide classroom student engagement opportunities [23,24,25]. Teachers often use questions to check students’ classwork, review or summarize lessons, diagnose students’ difficulties, and evaluate students’ learning [5,14,26,27,28]. For example, they are also used to determine what students have learned, to motivate and arouse student interest at the start or during the lesson, or to check students’ understanding of the concept during or at the end of a lesson. 

### 2.2. Technology and Teacher Questioning

The development of information technology has transformed all aspects of education and teaching. Teaching questioning, as one of the teaching tools, has also been affected. Big data technology and computer technology have become the mainstream technologies of classroom question analysis. Siti Khadijah Mohamad et al. [29] utilized innovative analytical analysis techniques, such as association rules mining, to determine how the provision of specific feedback through the questioning approach can trigger specific reflective thinking skills. Wang et al. [30] adopted the IRT modeling method to predict tendencies of teacher questioning in the process of classroom teaching. Ma et al. [31] used deep learning technologies, such as Convolutional neural network (CNN) and long short memory network (LSTM), to automatically analyze the content and type of teachers’ classroom questions. There are also some studies on the use of technology as a tool to improve teachers’ questioning skills. Lee and Yeo [32] developed an AI-based chatbot that engaged preservice teachers (PSTs) in an authentic, meaningful, and open-ended teaching situation to enhance PSTs’ responsive teaching skills, specifically questioning skills through approximations of practice.

### 2.3. Cognitive Diagnostic Model Application

In education, cognitive diagnostic theory is a new-generation measurement theory that combines cognitive and psychological measurements. Through learners’ observable assessment data, the cognitive diagnostic model (CDM) can analyze their comprehensive abilities and internal cognitive processing processes. It also comprehensively diagnoses their learning status from both macro and micro perspectives and assesses learners. It has attracted the attention of researchers and educators. Indeed, researchers have proposed several influential cognitive diagnosis models (CDMs) for different assessment scenarios. Representative works based on dichotomous scoring (zero or one) include the IRT model, the DINA model [33], the G-DINA model [34], and the HO-DINA model [35], which have been widely recognized and applied. Based on polytomous scoring scenarios, researchers have proposed P-DINA models, Fuzzy CDM [36], and so on, which can be used for subjective problem diagnosis and evaluation. Statistically, researchers have developed many types of CDMs for educators to apply to different education scenarios.

CDMs can diagnose learners’ abilities and knowledge structures in various educational settings, followed by remedial instruction. For example, Fischer [37] used the LLTM model to analyze the computational difficulty of participants in mathematical operations and conducted remedial teaching. Sandra Bley [38] used cognitive diagnostics to assess students’ skills and tasks and identify their strengths and weaknesses. It can also provide diagnostic results to teachers, thereby laying the foundation for effective teaching and personalized guidance. Fatemeh Ranjbaran et al. [39] developed a second language reading test based on a cognitive diagnostic model. The teacher used the test results to improve the teaching plan and provided rich second-language reading materials to meet the needs of students at different levels. Extensive empirical research has shown that applying cognitive diagnostic models in remedial teaching improves learners’ academic performance.

In this paper, we argue that more attention should be paid to how technology supports effective questioning by teachers as a starting point for developing productive dialogues. Although the importance of teacher questioning has been highlighted in this literature review, research on teacher questioning is still scarce and relatively outdated. We have not yet seen relevant research on technology as a tool to support personalized teaching questioning, especially in the application of cognitive diagnostic technology. This study explores the diagnosis of learners’ cognitive structure based on cognitive diagnostic techniques and proposes personalized questioning strategies based on the diagnostic results.

## 3. Theoretical Underpinnings

Cognitive structure refers to the knowledge structure in students’ minds, which is the content and organization of all concepts in students’ minds. It is an essential factor influencing learning and transfer. The characteristics of each cognitive structure in terms of content and organization are called cognitive structure variables, which mainly include usability, distinguishability, and stability. The “usability” of cognitive structure, that is, when students face new learning tasks, their cognitive structure should have the original concept of absorbing and fixing new knowledge to produce meaningful learning. The “distinguishability” of cognitive structure refers to the degree to which new learning tasks can be distinguished from the relevant knowledge by assimilating them. The “stability” of a cognitive structure refers to whether the existing knowledge in a student’s cognitive structure is stable and consolidated when facing new learning tasks. The original cognitive structure is influenced by these three variables in the learning of new knowledge. The theory of cognitive structure transfer points out that when students learn new knowledge, the higher the cognitive structure’s usability, distinguishability, and stability, the more it can promote the transfer of new knowledge learning.

According to the above theory, if we can clearly understand learners’ cognitive structures, we can change the state of their cognitive structural variables through questioning, making them highly useable, distinguishable, and stable. An excellent cognitive structure can promote learners’ knowledge, learning, and transfer.

## 4. PTQ Strategies Based on Learner’s Cognitive Structure Diagnosis

The PTQ strategies include learners’ cognitive structure diagnosis, personalized questions based on diagnostic results, and PTQ practice.

### 4.1. Learners’ Cognitive Structure Diagnosis 

**Figure 1:**
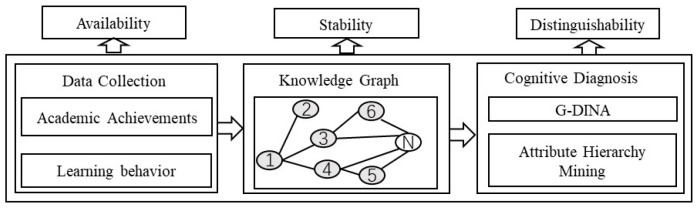
Learner’s cognition structure diagnosis. 1–6 in the Knowledge graph are the serial numbers of knowledge points.

Personalized and adaptive learning support services require an accurate assessment of the current learning status of students [40]. Diagnosing a learner’s cognitive structure is the basis for achieving personalized instructional delivery. The diagnosis of learners’ cognitive structures requires the use of personalized systems. Figure 1 shows the process of diagnosing learners’ cognitive structures. Firstly, the personalized system utilizes different information technologies to collect multimodal data (academic performance and learning behavior, etc.) generated during the teaching process in a comprehensive and automated manner. Secondly, based on disciplinary knowledge graphs and cognitive diagnostic techniques, the cognitive structure status of learners is diagnosed. The diagnostic results are represented by the knowledge structure’s usability, discriminability, and stability.

Usability represents the mastery state of subject knowledge in the learners’ original cognitive structure. It mainly uses learners’ performance scores on test items such as learning content, learning activities, and test questions as input. According to Bloom’s Taxonomy of Teaching Objectives, usability results can be expressed from low to high. High usability shows that the knowledge in the original cognitive structure of learners is in good condition and can assimilate or accommodate new knowledge. At the same time, there are two reasons for low usability. One is that learners have not yet mastered the relevant knowledge points, and the other is that the cognitive state of the knowledge does not meet the curriculum requirements.

Distinguishability refers to the degree of clarity between new and old knowledge, with results ranging from low to high. High distinguishability indicates that learners can accurately distinguish between old and new knowledge. Low distinguishability means that learners cannot accurately distinguish between old and new knowledge. One situation is that inadequate mastery of ancient knowledge leads to confusion in learning new knowledge; another situation is that new knowledge is not yet proficient, causing chaos between old and new knowledge.

Stability refers to learners’ proficiency in relevant knowledge within their original cognitive structure. It can be measured by the probability of the learner answering the same knowledge correctly. The deep knowledge tracking model can be used to explore the potential knowledge state of learners when continuously answering test questions. High stability means that the knowledge in the learner’s original cognitive structure and the correlation between the knowledge is stable. Low stability means loose connections between knowledge or lack of proficiency in certain knowledge points.

### 4.2. Personalized Questions Based on Diagnostic Results 

The theory of cognitive structure transfer posits that the greater the usability, distinguishability, and stability of learners’ cognitive structure, the more it facilitates the transfer of new knowledge when students learn new knowledge. The purpose of PTQ strategies is to compensate for the learner’s cognitive structure’s usability, increase the distinguishability of knowledge, and improve the stability of the cognitive structure, thus promoting the acquisition and transfer of new knowledge through strategically designed questioning at varying depths and time.

**Figure 2:**
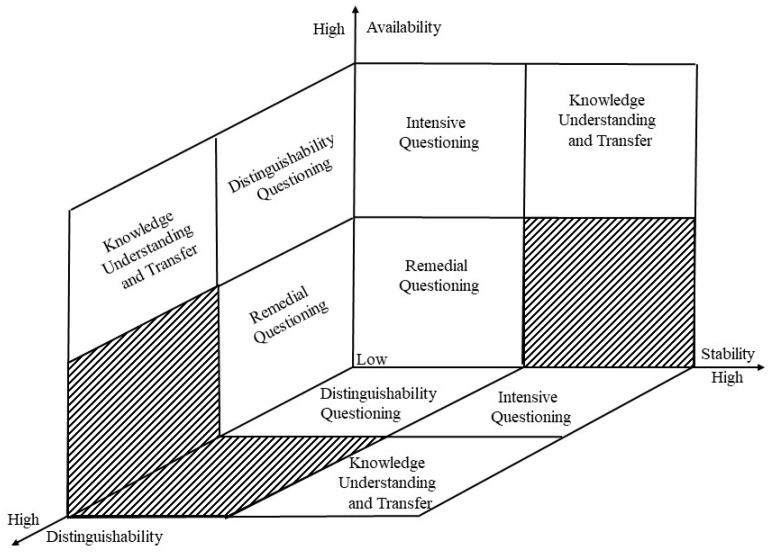
Personalized questions based on diagnostic results.

Figure 2 forms a three-dimensional coordinate axis using cognitive structural variables. The stability is represented on the horizontal axis, usability on the vertical axis, and the distinguishability of the cognitive structure on the oblique axis. All cognitive variables range from low to high. According to the theoretical support and teaching practice, the three-dimensional coordinate axis is divided into three zones. One is the perfect area. For learners in a perfect region, the values of the three cognitive structure variables are relatively high, and their cognitive structure can accommodate new knowledge, promoting the mastery and transfer of new knowledge. The second is a non-existent area. The non-existent area means that the cognitive structure of the learner does not exist in this situation. For example, learners’ cognitive structure can not be located in areas of high stability and low usability, as high stability and low usability are contradictory. The third region is typical for learners’ cognitive structure variables in teaching practice, with six situations.

1. When learners’ cognitive structure is of low availability and low distinguishability, it means that there are no prior knowledge points in their original cognitive structure that can accommodate new knowledge. They are not clear about identifying appositive knowledge points. Teachers should ask learners low-level, remedial questions at the beginning of the lessons to promote their understanding of existing knowledge and strengthen the usability of their cognitive structure. Raise higher-order questions about distinctiveness during the teaching process to increase the distinctiveness of new and old knowledge.

2. When the usability and stability of the learners’ original cognitive structure are low, it indicates that their original cognitive structure is relatively dispersed, and the proficiency level of relevant knowledge points is relatively low. Teachers should raise remedial questions at the initial stage of the curriculum to strengthen learners’ cognitive structures. At the same time, reinforcement questions are proposed in the cognitive state of learners towards knowledge points and in their nearest development zone to enhance the stability of learners’ cognitive structure.

3. There are two situations in which learners’ cognitive structures are of high usability and low stability. One is that learners are not proficient enough in the knowledge points, and teachers should propose intensive questions during the teaching process to enhance the stability of learners’ cognitive structure. Another situation is that the correlation between knowledge in the original cognitive structure is not yet stable, and distinguishable questioning can promote stability.

4. Learners with high usability and low distinguishability in their original cognitive structure lack mastery of new knowledge points. They cannot distinguish the differences and connections between new and old knowledge. According to Ausubel’s current organizer strategy, teachers can raise discriminative questions during the teaching process to help learners clarify the similarities and differences between new and old knowledge.

5. When the stability and distinguishability of learners’ cognitive structure are low, learners’ proficiency in knowledge points in the original cognitive structure is insufficient. When learning new knowledge points, it is easy to be confused by new knowledge. Therefore, teachers should put forward intensive questions based on the Zone of Proximal Development of the cognitive level of learners. Teachers should pay attention to putting forward discriminatory questions and assisting learners in establishing associations between new and old knowledge points.

6. When learners have high stability and low distinguishability, they are proficient in mastering the knowledge points in their original cognitive structure. During the teaching process, they should raise questions at different depths for new knowledge points to strengthen their mastery.

### 4.3. PTQ Practice

**Figure 3:**
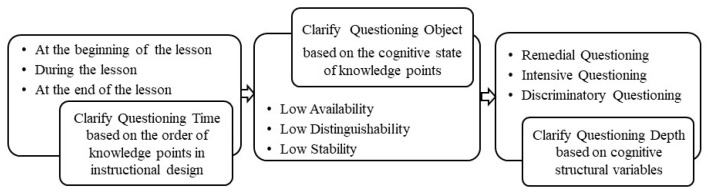
PTQ Practice.

Effective personalized questioning requires pre-planning regarding questioning time, depth, and objects. As shown in Figure 3, PTQ practice includes three aspects: clarifying the questioning time, the questioning object, and the question’s depth.

1. Clarify the questioning time based on the order of presentation of knowledge points in instructional design.

In the teaching design process, teachers present different knowledge at different teaching periods based on the learning situation of the class and the correlation between knowledge. Generally speaking, questioning is used to check what students have learned at the start or to check their understanding of the concept during or at the end of the lesson. When a teacher wants to know about a learner’s existing knowledge, he or she asks questions at the beginning of the lesson. Similarly, the teacher asks questions about new knowledge during or at the end of the lesson.

2. Clarify the questioning objects based on the knowledge pointing to the cognitive state of the learner.

Once the knowledge is identified, the teacher can determine the questioning object based on the state of the learners’ cognitive structural variables. Learners were selected as questioning subjects when their knowledge points did not meet the standards. For example, learners whose cognitive structural usability is not up to standard may be asked questions at the beginning of the course. Learners whose cognitive structural stability is not up to standard may be questioned throughout the course.

3. Clarify the depth of the question based on the cognitive structure variable state of learners.

Once the subject of the question is identified, the teacher can clearly understand the learner’s cognitive structure variable state through the results of the cognitive structure diagnosis. Teachers can conduct specific analyses of situations with low cognitive structural variables and propose appropriate depth questions (as described in Section 4.2). According to the theory, before any teaching takes place, the teacher will predict the knowledge state of the student(s), that is, to figure out their Zone of Proximal Development [41], which will help the teacher formulate an appropriate representation of the knowledge to be transmitted to the student(s). Questions create zones of proximal development when teachers are able to grasp their students’ prior knowledge, thinking, and inquiries to scaffold them to the next level. When the knowledge does not meet the standard, the teacher proposes the next in-depth question based on the learner’s current cognitive state of the knowledge point.

## 5. Methodology

### 5.1. Participants

At M Primary School in Ningxia, China, a math teacher and her class of fourth-grade students (n = 97, female = 45, male = 52) participated in this study. The teacher taught for over 20 years and was respected in her teaching communities. She showed a willingness to share and improve their practices. Before the experiment, the teacher learned how to use data and how to use questioning strategies to ensure that the experiment works. The experiment was carried out for two months.

### 5.2. Experimental Procedures

**Figure 4:**
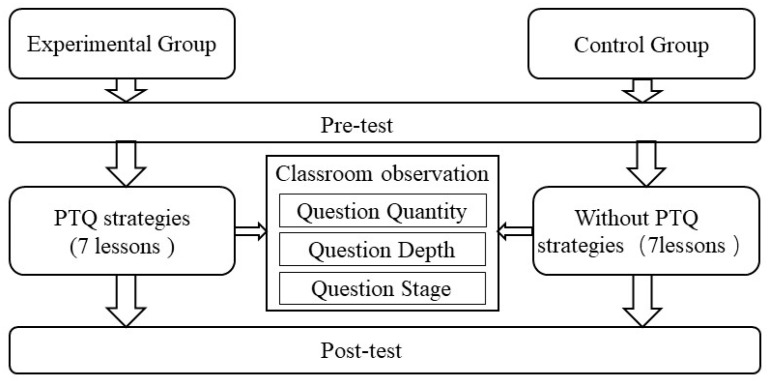
Experimental procedure.

The selected teaching content was Unit 6, Division by Two Digits, from the Human Education Edition textbook of Primary School Mathematics, Volume 1 of Grade 4. Seven lessons were given, each focusing on a specific knowledge point. Before the experiment, the two groups were pre-tested, and the experimental group underwent cognitive structure diagnosis based on the test results. The diagnostic results were used by the teacher to design personalized classroom questions. The entire questioning process was recorded and videotaped by the researchers. After the experiment, a post-test was conducted on both groups to evaluate the effectiveness of the personalized teaching questioning strategies based on cognitive structure diagnosis. The experimental procedure is shown in Figure 4.

### 5.3. Data Collection

Data collection mainly included four parts: pre-test scores, post-test scores, homework scores, classroom videotape, and classroom observation form. Data were collected through both automatic machine collection and manual collection methods. The learner’s homework scores, pre-test scores, and post-test scores were collected automatically by the extensive data system, while the classroom videotape was recorded automatically through the indoor recording and broadcasting system. The classroom observation form was collected by on-site observation by the researchers who entered the classroom. The homework tests, pre-test, and post-test used in the data experiment were jointly designed by four frontline teachers from the mathematics research group and two prominent data experts. The classroom observation form was to record questioning objects, questioning time, questioning numbers, and questioning depth during the teaching process through observation records. Classroom videos verify whether teachers follow personalized questioning strategies during the teaching process. They also provide additional verification for the collection of the teaching process.

## 6. Data Analysis

### 6.1. The Analysis of Pre-and Post-Test Data

The study compared the pre-test scores of two groups of students using independent sample *t*-tests. There was no significant difference between the pre-test scores of the two groups, indicating that their academic levels were at the same level. After the experiment, the post-test scores were compared between the two groups, and significant differences were found. The mean score of the experimental group (M = 93.3673) was significantly higher than that of the control group (M = 88.0833), indicating that the PTQ strategies based on cognitive structure diagnosis effectively improved learners’ academic performance. The research findings highlight the potential benefits of using the PTQ as a teaching strategy.

### 6.2. Time Series Analysis of Experimental and Control Groups

To gain deeper insights into the effectiveness of PTQ strategies based on cognitive structure diagnosis, the researchers conducted a time series analysis of eight experimental data from the experimental and control groups. As presented in Table 1, the research findings indicated that no significant improvement in the first four tests was observed. From the fifth experiment onwards, there was a significant difference in the scores of the experimental and control groups.

### 6.3. Comparative Analysis of Subject Literacy

**Figure 5:**
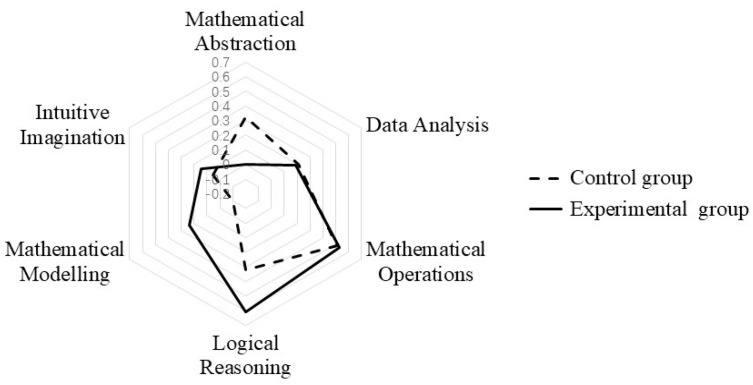
Comparative analysis of subject literacy in the experimental and control groups.

Mathematical core literacy includes six aspects: mathematical abstraction, data analysis, mathematical operations, logical reasoning, mathematical modeling, intuitive imagination. As shown in Figure 5, in terms of mathematical abstraction, the experimental group’s value was −0.001, much lower than the control group’s 0.323. In terms of data analysis, the value of the experimental group is 0.186, which is not significantly different from the control group’s 0.207. In terms of mathematical manipulation, both groups showed almost identical performance, with values of 0.5. In terms of intuitive imagination, logical reasoning, mathematical modeling, and intuitive imagination and reasoning, the experimental group’s level is much higher than that of the control group. The experimental group had values of 0.609, 0.233, and 0.144, while the control group had values of 0.323, −0.101, and 0.155, respectively. This discovery indicates that PTQ plays an important role in cultivating learners’ intuitive imagination, mathematical models, and logical reasoning, but has little effect on data analysis and mathematical operations. This strategy may have an inhibitory effect on cultivating learners’ mathematical abstraction.

### 6.4. Comparison of the Number of Questions Asked in the Experimental and Control Groups

**Figure 6:**
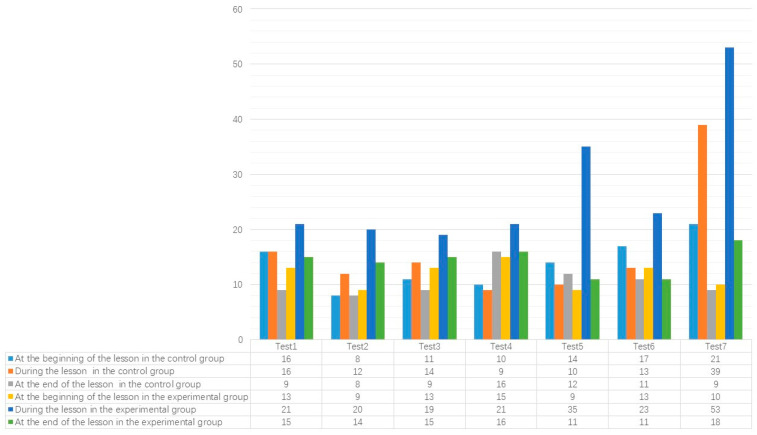
Shows the number of questions asked in the experimental and control groups at different times.

Figure 6 shows the impact of using a personalized teaching question PTQ strategy based on cognitive structure diagnosis on the total number of questions. The average number of questions per class in the experimental group was 54.8, higher than 40.5 in the control group. However, both of them were significantly higher than the average number of questions found in Cerry’s study (43.6). In the stage of teaching new knowledge, the number of questions in the experimental group was much higher than that in the control group, indicating that the PTQ strategy played an important role in promoting learners’ knowledge construction. At the end of the lesson course, which is the stage of strengthening knowledge and summarizing the lesson, the number of questions asked in the experimental class was higher than that in the control group. This indicates that after using the PTQ strategy, teachers’ emphasis on learners’ original cognitive structure decreased, shifting towards learners’ knowledge construction and transfer. 

### 6.5. Comparison of Question Depth

**Figure 7:**
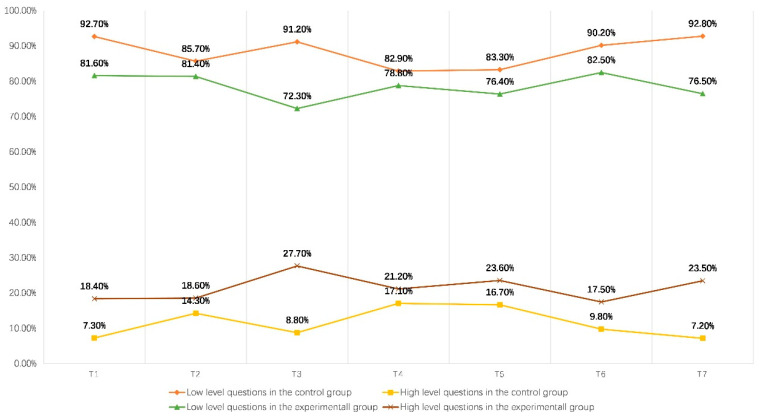
Number of questions asked by teachers at different levels.

Figure 7 shows the number of questions at different depths in the experimental and control groups. In the control group, 92.7% of the classroom questions were at a lower level of cognition, and the number of low cognitive problems was much higher than that of the experimental group. On the contrary, in terms of the number of high cognitive problems, the control group had a much lower number than the experimental group, with only 7.3%, which is consistent with previous research results [42,43,44,45]. This indicates that the PTQ strategy can enhance teachers’ questioning skills and help them raise high cognitive level questions in the classroom.

### 6.6. Distribution of Learners Asked in Class in the Control and Experimental Group

Related studies have showed that effective questioning can promote more learner engagement. Classroom observation was conducted to record their distribution to better understand the impact of PTQ strategies on teachers’ questioning objects. The study randomly selected the third lesson as a sample to examine the distribution of the questioning objects. Researchers used a horizontal and vertical coordinate table to show the distribution of respondents and only recorded the same person once if they were interviewed multiple times.

**Figure 8:**
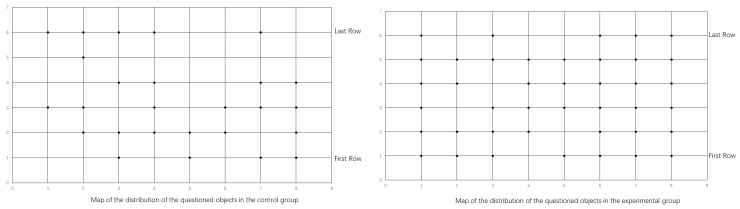
Distribution of learners asked in class in the control and experimental groups.

As shown in Figure 8, in the control class, the number of students being questioned was relatively small, and the positions of the students were concentrated in the first three rows of the class, which is the position of the teacher’s visual center. In the experimental class, there were a large number of students who were asked questions, and the location range almost covered the entire class. This indicates that the PTQ strategy can help teachers interact with students from different locations.

This study once again counted the number of times the same student was asked multiple times in order to explore the questioning habits of teachers. In the control class, nine people were asked multiple times in class, two-thirds of whom were outstanding students, and some were asked more than three times. In the experimental class, the number of respondents who asked multiple questions was reduced to four, and their academic performance was uneven. William [7] believes that this is likely to happen when teachers ask specific questions to a few random students who provide the answers they expect to hear.

## 7. Discussion

### 7.1. PTQ Strategies Based on Cognitive Structure Diagnosis

In this study, by leveraging big data and data mining, we can diagnose learners’ cognitive structures and propose PTQ strategies based on the diagnostic results. Compared to diagnosing the cognitive state, cognitive structure diagnosis more accurately reflects learners’ cognitive structure [46]. The description of the three cognitive structural variables can help teachers gain a more detailed understanding of the individual situations of learners. In addition, cognitive structure diagnosis technology, such as DINA, G-DINA, Logistic model, has played a role in providing services for personalized questioning, effectively integrating technology with the classroom. PTQ strategies solve the problem of providing personalized guidance for learners in classroom teaching and help teachers make their classroom discourse more thought-provoking. 

### 7.2. The Effectiveness ofPTQ Strategies Based on Cognitive Structure Diagnosis

1. The data indicate that PTQ strategies can effectively enhance learners’ academic performance, which is in line with previous research results such as Almeida et al. [1], Chin and Osborne [2], and Graesser and Olde [3]. Additionally, learners’ academic literacy improved correspondingly. Through a longitudinal analysis of relevant data, it was discovered that these strategies became effective after the fifth implementation, which may be attributed to the increased familiarity of both teachers and learners with the questioning strategies. 

2. In terms of teachers’ teaching behavior, PTQ strategies can increase the number of questions teachers ask. The average number of questions teachers ask in each class is 54.8, which is significantly lower than Schreiber’s approximately 64 questions per class. The research concluded that “the average number of questions asked by Chinese primary school mathematics teachers is 40”. Second, the proportion of higher-order problems in the experimental group reached 23.5%, much higher than the 5% found in Cunningham’s research 32 years ago, surpassing the 13% proportion found in dialogue and lecture classes [47]. However, current classroom questioning still tends to focus on lower-order questions. Some studies have also pointed out that this is related to teachers’ teaching beliefs that teaching mainly imparts knowledge through recall and repetition [48]. Although some attribute this inability to ask high-level questions to primary or secondary education [49], others attribute it to professional education from university [50]. Still, others attribute this inability to conventional habits of questioning and being questioned [45]. In order to reverse this trend, it is crucial to alter teachers’ beliefs and enhance their professional skills. In addition, related studies have pointed out that the depth of questions is related to question time and teaching content [13]. Before receiving personalized questioning strategy training, teachers can use low-level questions to strengthen learners’ original cognitive structures and enhance their usability. Teachers need to use higher-order questions to reinforcement learners’ cognitive structure during and after lessons, so as to improve their distinguishability and stability. PTQ strategies can also encourage more learners to participate in instructional questioning, with more than 80% of students receiving personalized questioning in each class, improving educational equity. 

## 8. Conclusions

This study shows that PTQ strategies based on learner cognitive structure diagnosis can effectively improve academic performance and subject literacy. Teachers’ professional skills have improved when using PTQ strategies, especially when asking in-depth questions. However, the applicability of these strategies to online teaching has yet to be experimentally verified. Further research is needed to explore how to propose personalized questions based on learners’ cognitive structure levels in online learning. This study highlighted the importance of carefully planning appropriate questions before implementation as well as the importance of understanding the cognitive structure of learners. Training teachers to ask higher-level questions appropriately is crucial; however, many teachers must be aware of this. We believe it is crucial to include this subject in the student–teacher curriculum and in-service teacher training programs so that teachers can be aware of the importance of classroom questioning and can be offered the strategies and tools to improve their questioning practices and, ultimately, improve their students’ learning experience.

## Figures and Tables

**Table 1:** Analysis of time series scores in experimental and control groups.

	Experimental Group		Control Group		T	p
	m	Sd	m	Sd		
Pre-test	96.9388	6.42070	94.1667	9.18679	1.725	0.088
Test1	98.0612	3.92857	96.4167	5.13160	1.770	0.080
Test2	86.3878	15.73719	83.6042	10.93450	1.010	0.315
Test3	88.3469	8.86837	90.3229	12.32300	−0.908	0.366
Test4	93.4694	8.72521	92.7917	9.94658	0.357	0.722
Test5	87.9592	10.21836	80.5833	21.49204	2.151	0.035
Test6	86.5918	9.87193	76.4375	13.67231	4.186	0.000
Test7	94.0816	5.92254	89.2708	14.43951	2.155	0.034
Post-test	93.3673	5.62618	88.0833	12.99932	2.589	0.012

## Data Availability

The datasets generated and analyzed during the current study are available from the corresponding author upon reasonable request.
